# Comparative Analysis of Gene Expression Data Reveals Novel Targets of Senescence-Associated microRNAs

**DOI:** 10.1371/journal.pone.0098669

**Published:** 2014-06-06

**Authors:** Marco Napolitano, Marika Comegna, Mariangela Succoio, Eleonora Leggiero, Lucio Pastore, Raffaella Faraonio, Filiberto Cimino, Fabiana Passaro

**Affiliations:** 1 IRCCS SDN Foundation, Naples, Italy; 2 Department of Molecular Medicine and Medical Biotechnologies, University of Naples Federico II, Naples, Italy; 3 CEINGE – Advanced Biotechnologies, Naples, Italy; University of Sassari, Italy

## Abstract

In the last decades, cellular senescence is viewed as a complex mechanism involved in different processes, ranging from tumor suppression to induction of age-related degenerative alterations. Senescence-inducing stimuli are myriad and, recently, we and others have demonstrated the role exerted by microRNAs in the induction and maintenance of senescence, by the identification of a subset of Senescence-Associated microRNAs (SAmiRs) up-regulated during replicative or stress-induced senescence and able to induce a premature senescent phenotype when over-expressed in human primary cells. With the intent to find novel direct targets of two specific SAmiRs, SAmiR-494 and -486-5p, and cellular pathways which they are involved in, we performed a comparative analysis of gene expression profiles available in literature to select genes down-regulated upon replicative senescence of human primary fibroblasts. Among them, we searched for SAmiR’s candidate targets by analyzing with different target prediction algorithms their 3’UTR for the presence of SAmiR-binding sites. The expression profiles of selected candidates have been validated on replicative and stress-induced senescence and the targeting of the 3’UTRs was assessed by luciferase assay. Results allowed us to identify Cell Division Cycle Associated 2 (CDCA2) and Inhibitor of DNA binding/differentiation type 4 (ID4) as novel targets of SAmiR-494 and SAmiR-486-5p, respectively. Furthermore, we demonstrated that the over-expression of CDCA2 in human primary fibroblasts was able to partially counteract etoposide-induced senescence by mitigating the activation of DNA Damage Response.

## Introduction

Described for the first time in 1961 by Hayflick and Moorhead [1] as a process that limited the proliferation of normal human cells in culture, cellular senescence currently refers to the essentially irreversible growth arrest that occurs when proliferating cells encounter a genotoxic stress and reveals a complex phenomenon incorporating both genetic and environmental components acting through convergent pathways. With the possible exception of embryonic stem cells [2], most division-competent cells, including adult stem cells and some tumor cells, can undergo senescence [3].

Cellular senescence is thought to have evolved as a mechanism to prevent that damaged DNA could be replicated and passed on to future generations of cells, thus being considered a tumor suppressor mechanism [3]. Nevertheless, despite their inability to replicate, senescent cells are metabolically active and develop an aberrant gene expression profile with proinflammatory behaviour, the so-called Senescence Associated Secretory Phenotype (SASP), that can induce or accelerate changes in normal surrounding tissues, explaining the possible implication in tumor promotion, aging and age-related pathologies [4]. Very recently, it has been reported that cellular senescence contributes also to embryonic development, both in mice and humans [5]–[6].

Distinctive features of senescent cells include enlarged and flattened morphology, the appearance of senescence-associated heterochromatin foci (SAHF), accumulation of senescence-associated DNA-damage foci (SDFs) and expression of Senescence-Associated β-galactosidase (SA-β-gal) [4].

Many senescence-inducing stimuli cause epigenomic disruption or genomic damage, like the gradual attrition of telomeres with each S phase [7], that generates a persistent DNA damage response (DDR), which initiates and maintains the senescence growth arrest of human cells both in culture and *in vivo* [8]–[9]. Persistent DDR signaling generated at nontelomeric sites also leads to the senescence growth arrest, such as that derived by strong mitogenic signals delivered by certain oncogenes or highly expressed pro-proliferative genes, that cause misfired replication origins and replication fork collapse [10]–[12]. Treatments with cytotoxic chemotherapeutic agents, such as etoposide, that cause DNA double strand breaks, also induce premature senescence via the p53 pathway [13]. Senescence can also occur, however, without detectable DDR signaling. These stresses could include inappropriate substrates or serum or oxidative stress, as the Reactive Oxygen Species (ROS) production after treatment with oxidative stress agents, such as the glutathione depletor Diethylmaleate (DEM) [14]–[15].

In the last years microRNAs (miRs) have added a new layer of complexity to the comprehension of molecular mechanisms underlying senescence. Each miR can recognize up to hundred different targets, thus influencing a large variety of cellular processes [16]. We and others have recently demonstrated that some miRs are involved in the process of cellular senescence [17]–[20]. In particular, we have found a subset of five Senescence-Associated miRs (SAmiRs) up-regulated during Human Diploid Fibroblasts (HDFs) replicative senescence, whose ectopic expression in young cells promoted the premature senescence program by inducing DNA damage and ROS accumulation.

The main aim of this study has been the identification of novel mRNA targets of two selected SAmiRs, SAmiR-486-5p and SAmiR-494. We chose SAmiR-486-5p, as we previously showed that this miR was the most robust up-regulated upon replicative senescence and significantly up-regulated upon Etoposide-Induced Senescence (EIS) of HDFs and in human primary skin fibroblasts from old donors [17]. Furthermore, Kim and colleagues demonstrated that the over-expression of SAmiR-486-5p was able to induce premature senescence also in human adipose tissue-derived stem cells, by inhibiting SIRT1 [21]. We also chose SAmiR-494, as its over-expression seemed to better recapitulate all the features of a senescent phenotype, including reduced cell proliferation, induction of SA-β-gal, SAHFs, DNA damage, SDFs and ROS accumulation. Moreover, it resulted up-regulated also in DEM - Induced Senescence (DIS) [17] and its over-expression was able to induce senescence not only in HDFs, but also in cancer cells [22].

Our strategy has been to look for new SAmiR targets among the mRNAs down-regulated in HDF senescent cells, comparing six different gene expression profiles available in literature, selecting genes whose expression was reduced in at least three different arrays and searching for SAmiR’s responsive elements into their 3’UTR by the use of different target prediction algorithms.

This approach allowed us to identify Cell Division Cycle Associated 2 (CDCA2) and Inhibitor of DNA binding/differentiation type 4 (ID4) as novel targets of SAmiR-494 and SAmiR-486-5p, respectively. Moreover, we have also demonstrated that the transient over-expression of CDCA2 in HDFs is able to partially counteract the premature senescent phenotype induced by etoposide treatment.

## Materials and Methods

### Bioinformatics analysis

We took advantage of available data reporting gene expression profiles in replicative senescent HDFs. Normalized data from 6 microarrays [23]–[28] have been crossed in order to select a list of common genes down-regulated in senescence, with a fold variation≥1.5. We obtained a list of 139 genes down-regulated upon HDFs replicative senescence (Table S1). Functional annotations were obtained on *PubMed.gov*. For the identification of putative targets of SAmiR-494 or SAmiR-486-5p, the genes in the list have been analyzed with four different target prediction algorithms (Target Scan v6.2, miRDB, Diana, miRanda) and putative targets predicted by at least two algorithms have been selected for further studies.

### Real time PCR

Total RNA was extracted with TRIzol Reagent (Life Technologies) and quantified by Nanodrop (Thermo Scientific, Wilmington, DE). The first-strand cDNA was synthesized according to the manufacturer’s instructions (SS VILO Mastermix- Life Technologies). Real-time RT-PCR was carried out on an iCycler (BioRad) using Express Greener QPCR Master mix (Life Technologies). The housekeeping beta-actin (ACTB) mRNA was used as an internal reference gene for normalization. PCR reactions were performed on biological duplicates or triplicates and in experimental triplicate. Fold changes were calculated using 2^−ΔΔ^ *Ct* method, by the formula: 2^−(sample ΔCt - calibrator ΔCt)^, comparing results from experimental samples (Replicative senescent cells, Etoposide-induced senescent cells or DEM-induced senescent cells) with both a calibrator (young PDL 33 cells for RS; DMSO treated cells for EIS and DIS) and the reference gene ACTB. ΔCt is the difference between the amplification fluorescent thresholds of the gene of interest and ACTB. The list of the primers used is reported in Table S2.

TaqMan MiRNA Assay Kit (Applied Bio-systems, Foster City, CA) was used to detect the expression of mature miRNAs. Briefly, 100 ng of total RNA was reversely transcribed (RT) at 16 °C for 30 min, 42 °C for 30 min and 85 °C for 5 min in 15 µl reaction volume. Two µl of RT product were used for PCR reaction in a final volume of 20 µl. The PCR reaction started with an initial denaturation step at 90 °C for 10 min, followed by 40 cycles of 95 °C for 15 sec and 60 °C for 1 min. Small nucleolar RNA RNU6 (Applied Biosystems, Foster City, CA) was used for normalization. PCR reactions were performed in triplicate and fold changes were calculated using 2^−ΔΔ^ *Ct* method, where ΔCt is the difference between the amplification fluorescent thresholds of the miRNA of interest and the RNA of RNU6.

### Cell cultures, treatments and transfections

Normal human primary fibroblasts IMR90 and human embryonic kidney HEK-293 cells were obtained from American Type Culture Collection (Manassas, VA). Cells were cultured in Dulbecco's modified Eagle's medium (DMEM) supplemented with 10% (v/v) fetal bovine serum and 1% penicillin/streptomycin (Gibco). Cultures were maintained at 37 °C in a 5% CO_2_-humidified atmosphere.

The IMR90 population doubling level (PDL) was calculated by using the formula: ΔPDL = log(*n_h_*/*n_i_*)/log2, where *n_i_* is the initial number of cells and *n_h_* is the final number of cells at each passage. The cells were used at 33 PDL (young) or 58 PDL (senescent) (Fig.S1).

To induce premature senescence, IMR90 at PDL 33 were treated with 150 µM DEM on alternate days for 10 days or with 20 µM etoposide (both from SIGMA-ALDRICH) for 24 h and then subcultured for 10 days more (Fig.S1).

Transfection of IMR90 cells at PDL 33 with synthetic pre-miR precursors (Ambion), miR inhibitors (Exiqon) or siRNAs (Dharmacon) were performed using Lipofectamine RNAiMAX Transfection Reagent (Life Technologies) with the reverse protocol following the manufacturer’s instructions. Pre-miRs, miR inhibitors and siRNAs were used at 100 nM.

Transfection of IMR90 cells at PDL 33 with CMV-CDCA2 and CMV-ID4, as well as co-transfection of HEK-293 with both luciferase constructs and pre-miRs were performed using Lipofectamine 2000 (Life Technologies) following the manufacturer’s instructions.

### Plasmid construction

With the exception of ID4, for which it has been cloned a portion of 300 bp containing the putative SAmiR-486-5p binding site, the whole 3’UTRs sequences of CDCA2, FOXM1, NUSAP1 and BUB1b have been cloned by PCR amplification on human genomic DNA, using primer pairs with XhoI and SalI restriction enzyme sites in the forward primers and XbaI in the reverse primers. A 430 bp portion of the 3’UTR of human OLFM4, containing the validated SAmiR-486-5p binding site [29], was cloned as positive control using primer pairs with SalI restriction enzyme sites in the forward primers and XbaI in the reverse primers. All PCR products were cloned into the pMIR-GLO vector (Promega) between the XhoI and the XbaI site, downstream the Firefly luciferase (Normal clones). The inverted 3’UTR of CDCA2, ID4 and OLFM4 (Reverse clones) were cloned by digestion of Forward clones with SalI, that allowed the excision of the 3’UTR, and by recloning digested fragment in SalI unique site. The orientation of the inserted fragments were established by digestions and confirmed with sequencing. The 3’UTRs of CDCA2 and ID4 containing point mutations in the SAmiR seed region (Mutated clones) were obtained by PCR using the Quik Change II XL site direct mutagenesis kit (Agilent), following the manufacturer’s instructions. Mutations were confirmed by sequencing.

The coding sequences of CDCA2 and ID4 were amplified by PCR from ULTIMATEHORF CLONE ID IOH44066 and ULTIMATEHORF CLONE ID IOH12413 (Life Technologies), respectively, and cloned into the pEGFPN1 vector, in place of the GFP coding sequence, between the AgeI and NotI sites. The obtained vectors were named CMV-CDCA2 and CMV-ID4. All primers used for plasmids construction and the oligos containing the mutated SAmiR seed regions are reported in Table S3.

### Luciferase Reporter Assay

For luciferase assays, human HEK-293 cells were plated at 8×10^4^ cells per well on 48 well plates (BD Falcon) 12 h before transfection. The Normal, Reverse or Mutated luciferase constructs (100 ng) were co-transfected with 50 nM pre-miRs. All transfection experiments were done in triplicate and each experiment was repeated three times. The Renilla luciferase reporter, contained into the pMIR-GLO vector, was used as an internal control. The luciferase activity was measured 48 hours after transfection using a Dual Luciferase Reporter Assay System (Promega) according to manufacturer’s instructions, on a 20/20^n^ Luminometer instrument (Turner BioSystems). The data generated were expressed as relative to control-miR transfected cells, after normalization to *Renilla* luciferase reading.

### SA-β-gal assay and BrdU assay

SA-β-gal was assayed according to Dimri *et al.* [30]. Briefly, cells were washed twice with PBS, fixed with 2% formaldehyde and 0.2% glutaraldehyde in PBS, and washed twice in PBS. Then, cells were stained overnight in X-gal staining solution [1 mg/ml X-gal, 40 mM citric acid/sodium phosphate (pH 6.0), 5 mM potassium ferricyanide, 5 mM potassium ferrocyanide, 150 mM NaCl, 2 mM MgCl_2_] at 37°C.

For BrdU (5-bromo-2-deoxyuridine) incorporation assay (ROCHE), cells were seeded on glass coverslips and transfected with siRNAs to induce the knock-down of SAmiR targets, or transfected with CMV-CDCA2, CMV-ID4 or CMV-NEO (as control plasmid) and treated with 20 µM etoposide to induce EIS or with 150 µM DEM to induce DIS. 72 h after transfection (siRNAs) or 24 h after treatments (48 h after plasmids over-expression), cells were incubated for 4 h with BrdU (10 µM) and fixed following the kit instructions. Coverslips were incubated with a primary anti-BrdU and a secondary fluorescein-conjugated antibodies and then counterstained with Hoechst 33258, rinsed and mounted in Moviol on glass slides. The fluorescent signal was visualized with an epifluorescent microscope Leica DM IL LED FLUO (Leica Microsystems). At least 300 cells were counted in triplicate experiments.

### Immunofluorescence

To perform γ-H2AX staining, IMR90 at PDL33 were plated on glass coverslips, transfected with CMV-NEO control vector or CMV-CDCA2 for 24 h and, then, treated with 20 µM etoposide. Coverslips were collected at 0 h, 6 h and 18 h after treatment, fixed with 4% paraformaldehyde in PBS for 30 minutes at RT, permeabilized with 10% FBS, 1% BSA, 0,2% Triton in PBS for 15 minutes at RT and incubated with Anti-phospho-Histone H2A.X Ser 139 primary antibody (Millipore) for 2 h at RT. After 4 washes of 5 minutes each, coverslips were incubated with an Alexa-488 goat anti-mouse antibody (Life Technologies) for 1 h at RT, counterstained with DAPI and mounted in Moviol on glass slides. Samples were observed with an epifluorescent microscope Leica DM IL LED FLUO (Leica Microsystems) and at least 300 cells were counted in triplicate experiments.

### Western blotting

IMR90 cells were harvested following washing with PBS. Cells were lysed in a buffer containing 0.02M HEPES (pH 7.9), 0.4M NaCl, 0.1% NP-40, 10% (v/v) glycerol, 1 mM NaF, 1 mM sodium orthovanadate and a protease inhibitory cocktail (Sigma Chemical Co. St. Louis, MO). Extracts were subjected to Sodium Dodecyl Sulfate (SDS)-polyacrylamide gel electrophoresis, followed by blotting to PVDF. The blots were probed with antibodies from Santa Cruz to human CDCA2, ID4, p53, p21, β-actin and α-tubulin; from Cell Signaling to human phospho-ATM (Ser 1981) and phospho-p53 (Ser 15) ; from Sigma-Aldrich to human ATM.

### Statistical analysis

Statistical analysis were carried out using the Student’s t test and data were considered significant at a value of p<0.05.

## Results

### A set of mRNAs is down-regulated in human senescent fibroblasts

**Figure 1:**
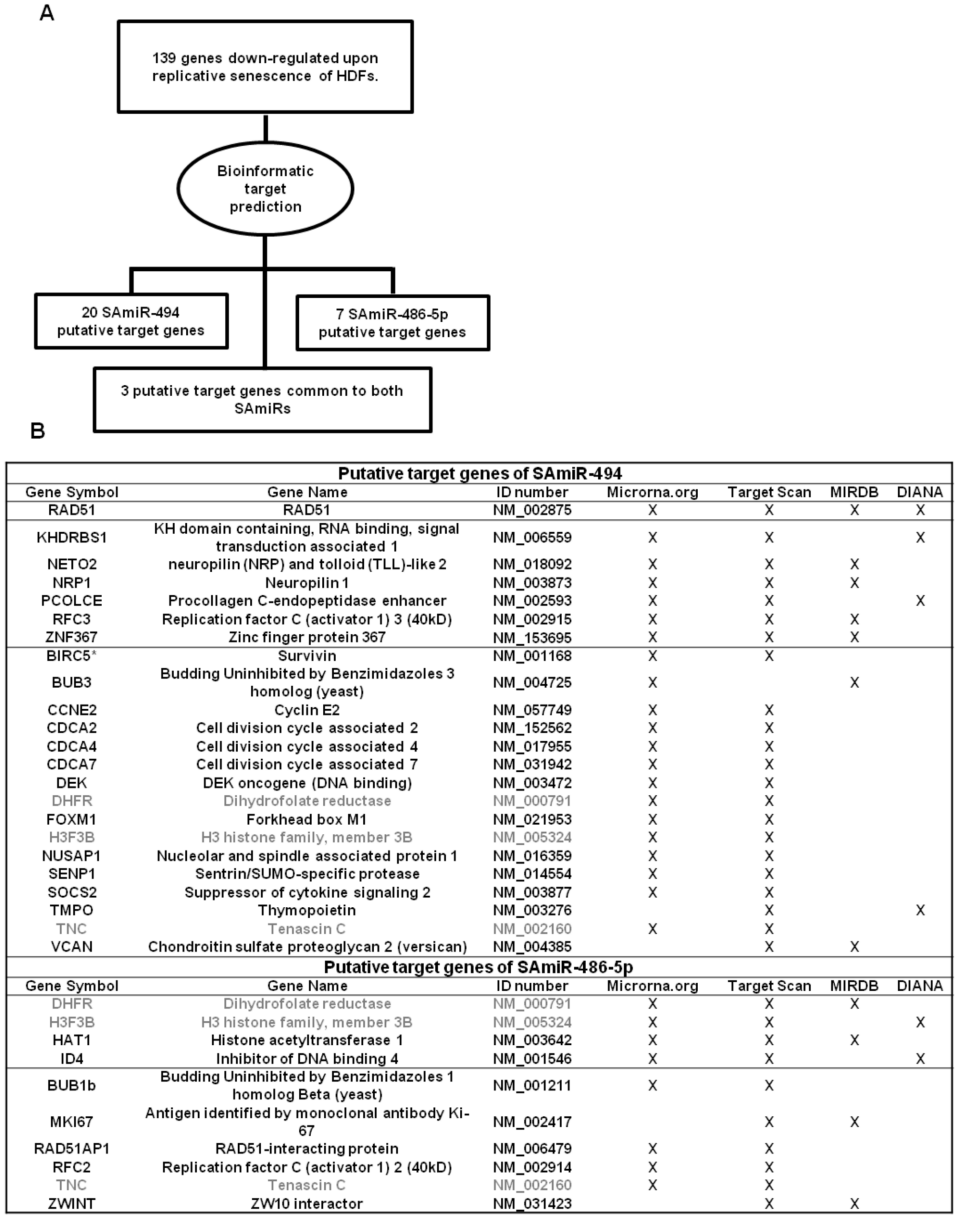
Strategy to identify putative targets of SAmiRs. A) The 3’UTR sequences of the 139 mRNAs down-regulated in replicative senescent fibroblasts and reported in Table S1 were analyzed with four different target prediction algorithms. This *in silico* analysis revealed 30 putative SAmiR targets: 20 of SAmiR-494, 7 of SAmiR-486-5p and 3 common to both SAmiRs. B) List of the 30 predicted target genes of both SAmiR-494 or SAmiR-486-5p. In grey, the three putative targets common to both SAmiRs.

To select candidate targets of SAmiR-494 or SAmiR-486-5p, we speculated that SAmiRs induced upon Replicative Senescence (RS) of HDFs could contribute to the suppression of genes that must be kept down-regulated on the induction of RS. Thus, we generated a list of mRNAs down-regulated on HDFs senescence by comparing the normalized data of six different microarray gene expression profiles available on public databases [23]–[28]. This analysis allowed to select 139 mRNAs down-regulated(≥1.5 folds) in at least 3 out of 6 different arrays (Table S1). As summarized in Fig. 1A, we analyzed the 139 mRNAs for the presence of consensus motifs for SAmiR-494 and/or for SAmiR-486-5p, by using four different target prediction algorithms (Target Scan v6.2, miRDB, Diana, miRanda) and focusing on the results common to at least two algorithms. This screening led to the generation of the list of candidate targets shown in Fig. 1B, with 20 putative targets of SAmiR-494, 7 of SAmiR-486-5p and 3 common to both SAmiRs (in grey). Among them, there are many mRNAs encoding proteins involved in cell cycle regulation (e.g. CCNE2, NUSAP1, ZWINT) and DDR (e.g. RAD51, RAD51AP1, DEK), two biological processes modified by ectopic expression of SAmiRs [17] (see also Table S1 for functional annotations). Interestingly, some of the candidates are members of the same protein family (e.g. BUB3 and BUB1b; CDCA2, CDCA4 and CDCA7; RFC2 and RFC3). We excluded BIRC5 (survivin) from further investigation, as it was already validated by others [31].

**Figure 2:**
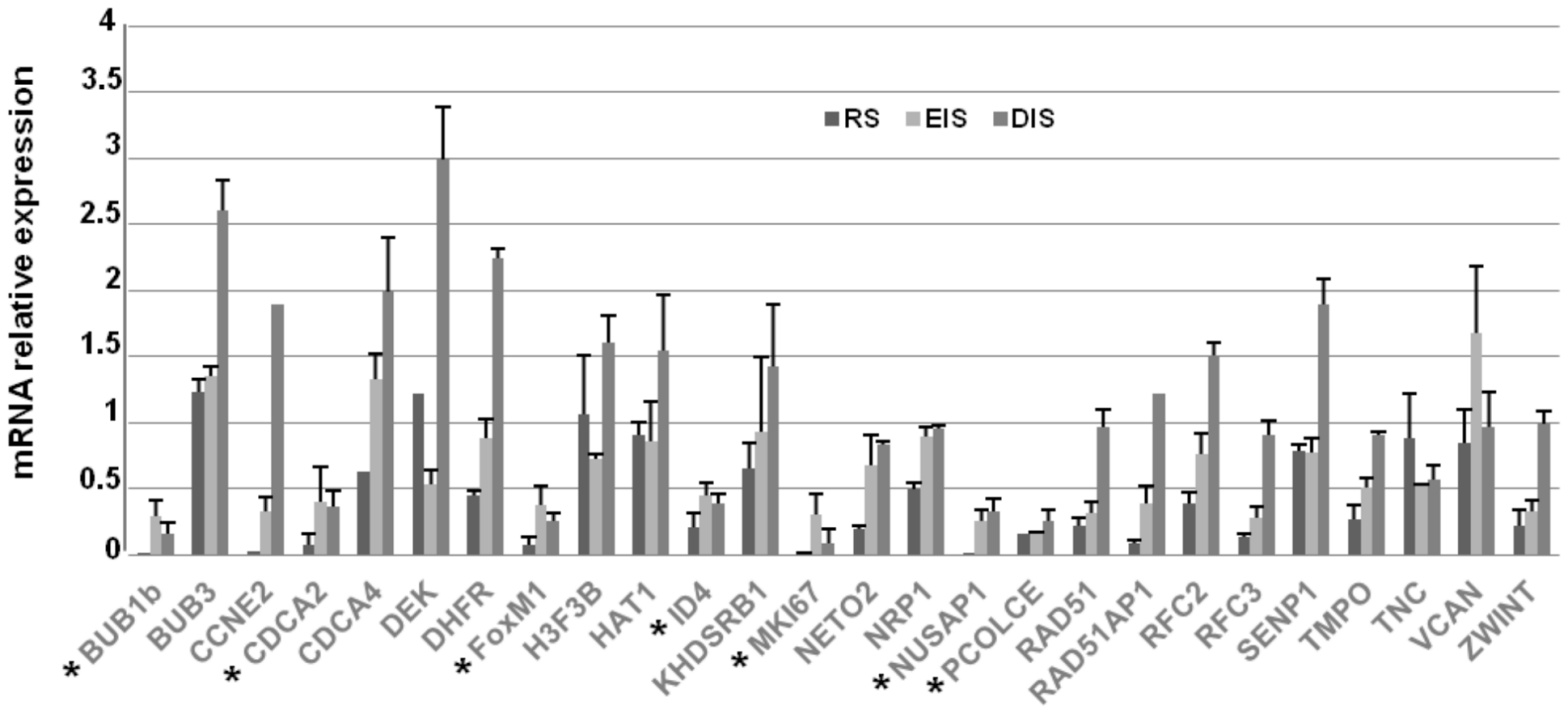
Expression profile of putative targets upon the induction of replicative or stress-induced senescence. The expression levels of the 26 candidates were measured by Real Time PCR in replicative (**RS**: IMR90 cells at PDL 58), etoposide- (**EIS**: PDL 33 IMR90 cells treated with 20 µM etoposide for 24 h and then subcultivated for additional 10 days) and DEM-induced (**DIS**: PDL 33 IMR90 cells treated with 150 µM DEM on alternate days for 10 days) senescent cells. The mRNA relative expression was calculated by assigning the arbitrary value 1 to the amount found in young or DMSO-treated cells. SD is used to refer to the values obtained in 2 different experiments. Results showed that 7 mRNAs, highlighted by a star, resulted significantly (p<0.05) down-regulated, with a cut-off≥2 folds, in all conditions.

To validate the results of our comparative analysis, we investigated in IMR90 cells the expression profile of putative targets upon induction of RS, EIS and DIS (Fig. 2). With the exception of CDCA7, SOCS2 and ZNF367, whose expression in IMR90 was undetectable (not shown), the results obtained by Real Time PCR demonstrated a common signature of gene expression in replicative and stress-induced senescence, with 17 out of 26 putative target genes that resulted significantly (p<0.05) down-regulated, with a fold variation≥2, upon RS, 14 out of 26 upon EIS and 7 out of 26 upon DIS. These results prompted us to select for further investigation the 7 candidates, highlighted in Fig. 2 by a star, whose expression resulted down-regulated in all the examined conditions (RS, EIS and DIS).

### A subset of putative targets are down-regulated upon SAmiR over-expression in human fibroblasts

**Figure 3:**
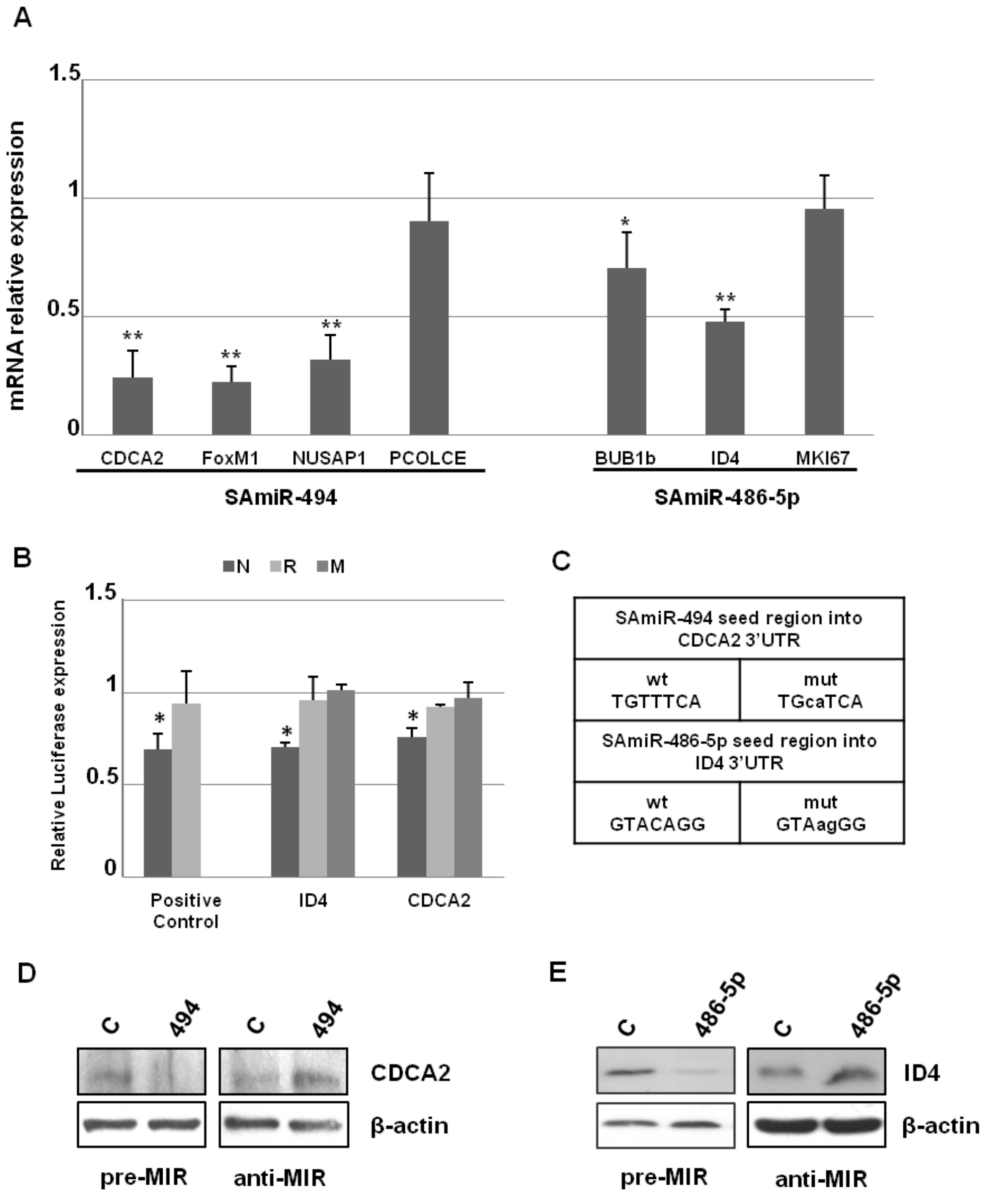
Expression profile of putative targets upon SAmiRs ectopic expression and validation of CDCA2 and ID4. A) Expression levels of SAmiR-494 and SAmiR-486-5p putative targets 2 days after the transfection of the cognate SAmiR pre-miR in PDL 33 IMR90 cells. mRNA levels were measured by Real Time PCR and mRNA relative expression was calculated by assigning the arbitrary value 1 to the amount found in control cells transfected with a scramble pre-miR. SD refers to the values obtained in 3 different experiments and the difference was significant (** p<0.01; * p<0.05). The quantification of the expression levels of SAmiRs after their ectopic over-expression is reported in Panel A of Figure S2. B) Luciferase constructs bearing the normal 3’UTRs (N), reverse 3’UTRs (R) or mutated 3’UTRs (M) of CDCA2 and ID4 were transfected in HEK293 cells together with the cognate pre-miR or control pre-miR. The normal and reverse 3’UTR of OLFM4 were used as positive control. Luciferase levels were reported as fold changes compared to the values measured in control pre-miR transfected cells, after normalization with Renilla luciferase activity. SD refers to the values obtained in 3 different experiments (* p<0.01). C) Wild type seed regions of SAmiR-494 and SAmiR-486-5p, respectively present into the 3’UTRs of CDCA2 and ID4, compared to the mutated seed regions used for luciferase assays. D) Western blot analysis of CDCA2 in IMR90 cells over-expressing SAmiR-494 (pre-miR) or a specific SAmiR-494 inhibitor (anti-miR). E) Western blot analysis of ID4 in IMR90 cells over-expressing SAmiR-486-5p (pre-miR) or a specific SAmiR-486-5p inhibitor (anti-miR). In both cases, the proteins resulted suppressed by the SAmiR over-expression, as well as they resulted up-regulated by the anti-miR transfection, if compared to the control scramble transfected cells. The quantification of the expression levels of SAmiRs after their ectopic over-expression in D and E is reported in Panel B of Figure S2.

In order to identify direct targets of SAmiRs, considering that mRNAs targeted by a miR are generally degraded [32]–[33], we analyzed by Real Time PCR the mRNA levels of BUB1b, CDCA2, FOXM1, ID4, MKI67, NUSAP1 and PCOLCE at day 2 after the transfection of the cognate synthetic SAmiR precursor (pre-miRs). As shown in Fig. 3A, CDCA2, FOXM1 and NUSAP1 resulted significantly (p<0.01) down-regulated 2 days after SAmiR-494 pre-miR transfection, whereas, among SAmiR-486-5p putative targets, ID4 and, at a lower extent, BUB1b, showed a significant reduction upon pre-miR transfection.

### CDCA2 and ID4 are direct target of SAmiR-494 and SAmiR-486-5p, respectively

To address whether CDCA2, FOXM1, NUSAP1, ID4 and BUB1b, whose mRNAs resulted reduced after SAmiR over-expression, were direct targets, luciferase constructs containing their 3′ UTR sequences were generated. As shown in Fig. 3B, in the case of CDCA2 and ID4 the reporter gene expression was significantly reduced by SAmiR-494 or SAmiR-486-5p pre-miR transfection, respectively, with a variation of relative luciferase expression similar to the positive control OLFM4 (N). In contrast, the co-transfection of FOXM1, NUSAP1 or BUB1b luciferase constructs with the cognate SAmiR didn’t show any change in luciferase expression compared to unrelated pre-miR transfected cells (Fig. S3A).

To further characterize the functionality of predicted target sites in the 3’UTRs of CDCA2 and ID4, the corresponding reverse fragments or the fragments mutated at the seed region of putative target sites were also generated. As shown in Fig. 3B, the luc-reverse constructs (R), as well as the constructs bearing mutated UTRs (M) were unaffected by the cognate SAmiR transfection (see also Fig. 3C for point mutations into SAmiRs seed regions), thus strongly suggesting that CDCA2 and ID4 were direct targets of selected SAmiRs.

We also investigated the expression profiles of the two targets by Western blot analysis, upon SAmiRs over-expression or down-regulation in PDL 33 IMR90 cells. As shown in Fig. 3D and 3E, the decrease in CDCA2 and ID4 endogenous expression levels was well detectable at protein level. Noteworthy, the transfection of miR inhibitors caused the increase of target expression levels.

All together, these data demonstrated that CDCA2 and ID4 are direct targets of SAmiRs-494 and SAmiR-486-5p, respectively.

### Knock-down of CDCA2 or ID4 in young human primary fibroblasts does not induce senescence

**Figure 4:**
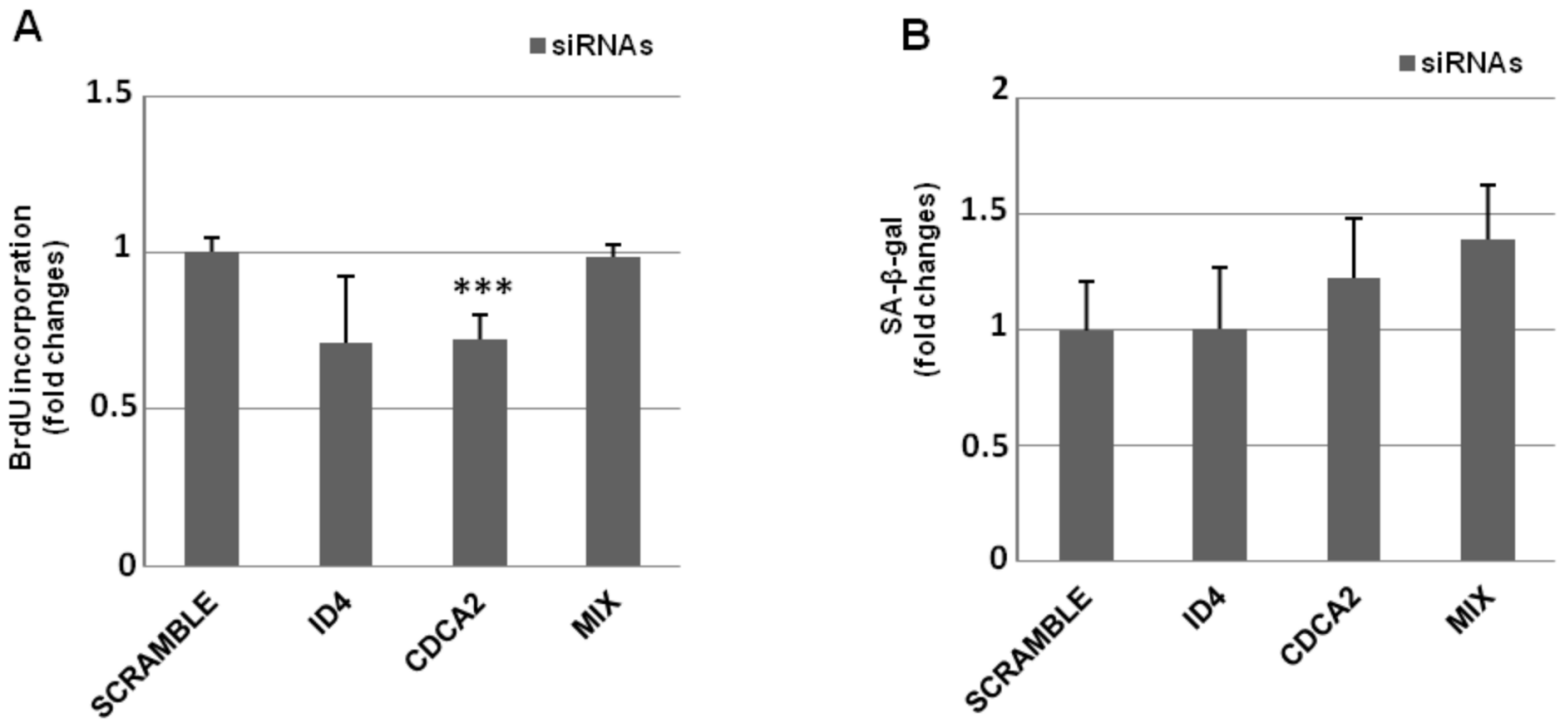
Knock-down of CDCA2, ID4 or both does not induce premature senescence in PDL 33 IMR90 cells. A) siRNAs designed to target the coding sequence of CDCA2 or ID4 were transfected, individually or as a mix, in PDL 33 IMR90 cells to knock-down the expression levels of target genes. After 72 h, transfected cells were incubated with BrdU for 4 h, then coverslips were fixed, incubated with a primary anti-BrdU antibody, washed and incubated with a secondary fluorescein-conjugated antibody, counterstained with Hoechst-33258 and counted by immunofluorescence. Counts of at least 1,000 cells were averaged and expressed as fold changes±SD, with respect to scrambled transfected cells (*** p<0.001). B) siRNA transfected cells were subcultivated for 10 days and stained for SA-β-gal. Counts of at least 300 cells were averaged and expressed as fold changes±SD, with respect to scrambled transfected cells.

To analyze the role of CDCA2 and ID4 in senescence, we asked whether their knock-down by RNAi in young cells was able to induce premature senescence, as the up-regulation of the cognate SAmiRs does. To this aim, we transfected siRNAs designed to silence CDCA2 or ID4 in young IMR90 at PDL 33 individually or as a mixture (Fig. S3B), and then we analyzed the cells until ten days after transfection, in order to detect any signs of senescence, as the decreasing of cell proliferation by BrdU incorporation, the change in cell morphology or the appearance of SA-β-gal. As showed in Fig. 4A, the knock-down of these genes seemed to be unable to affect cell proliferation, although a weak but significant decrease in BrdU incorporation was detected in siCDCA2 cells. Accordingly, knock-down cells did not senesce prematurely, as demonstrated by the absence of SA-β-gal staining 10 days after siRNA transfection (Fig. 4B). Probably, neither the transient down-regulation of individual SAmiR target, nor the knock-down of both targets have the “strength” to switching-on the senescence program, as the contemporaneous reduction of many targets exerted by SAmiR’s over-expression do.

Nevertheless, the down-regulation of CDCA2 or ID4 caused by the up-regulation of cognate SAmiRs could contribute to the acquisition of the final senescence phenotype. Thus, we investigated the possible role of CDCA2 or ID4 in cellular senescence by determining the effects of their over-expression.

### Adoptive expression of CDCA2 promotes cell cycle progression in EIS

We transfected CDCA2 or ID4 in young PDL 33 IMR90 cells, one day before the induction of EIS or DIS. Then, we monitored the progression of senescence by checking for BrdU incorporation, cell morphology changes and appearance of SA-β-gal.

**Figure 5:**
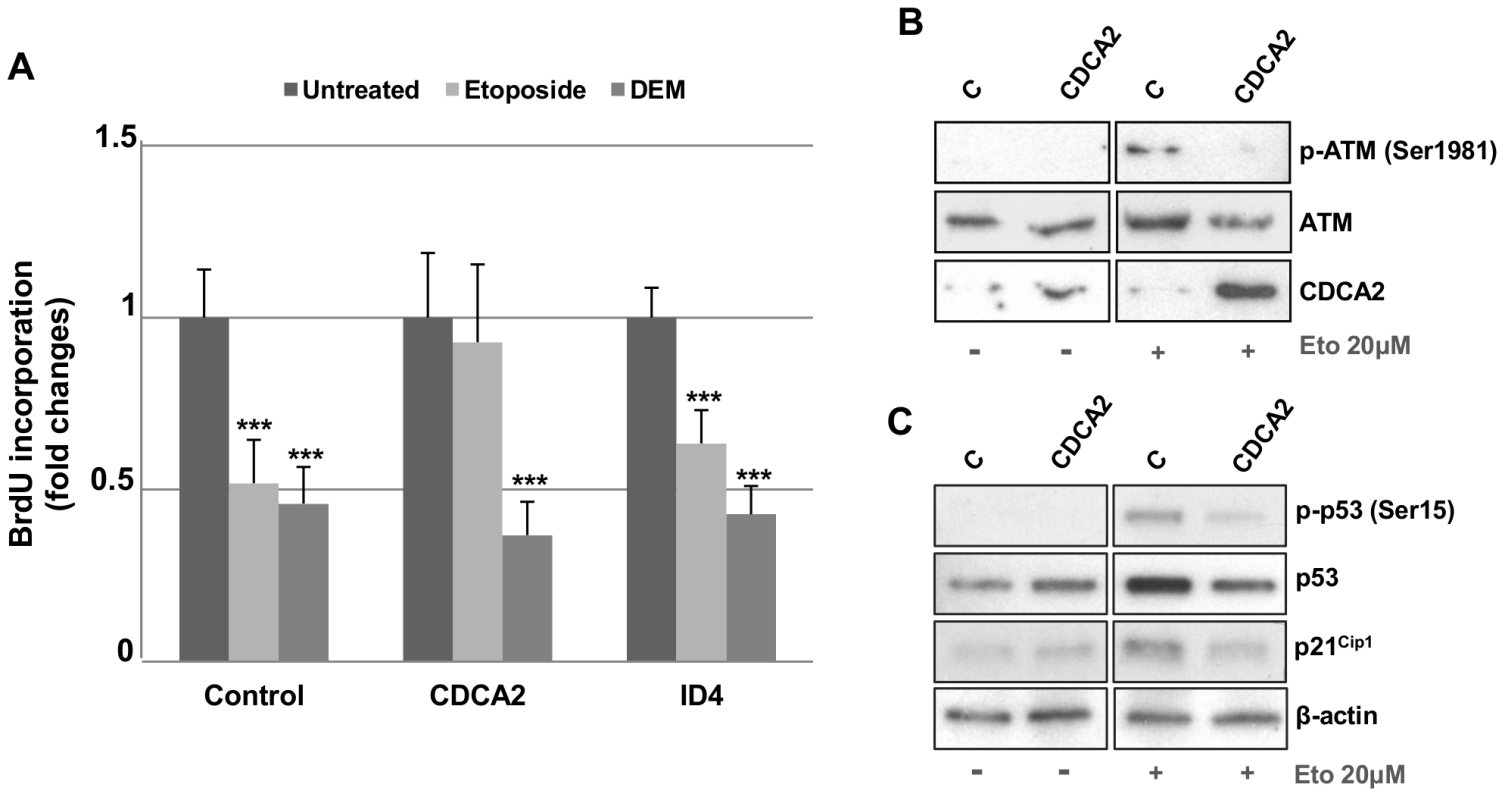
Adoptive expression of CDCA2 promotes cell cycle progression in Etoposide-Induced Senescence. A) CDCA2 and ID4 coding sequences were transfected in PDL 33 IMR90 cells. Cells transfected with CMV-NEO plasmid were used as control. After 24 h, transfected cells were treated with 20 µM etoposide or 150 µM DEM for 24 h and then incubated with BrdU for 4 h. Coverslips were then fixed, incubated with primary anti-BrdU and secondary fluorescein-conjugated antibodies, counterstained with Hoechst-33258 and counted by immunofluorescence. Counts of at least 800 cells were averaged and expressed as fold changes±SD, with respect to control transfected cells (***p<0.01). B) and C) PDL 33 IMR90 cells were transfected with the coding sequence of CDCA2. After 24 h, transfected cells were treated with 20 µM etoposide for 24 h and then were collected to obtain protein extracts. Cell extracts from CMV-neo over-expressing cells served as control. Western Blot analysis was used to detect the levels of phosphorylated ATM (p-ATM Ser1981), ATM, phosphorylated p53 (p-p53 Ser15), p53, p21^Cip1^ and CDCA2 in the cell lysates. β-actin was used as a loading control.

While DIS program seemed to be unaffected, the transient over-expression of CDCA2, but not ID4, was able to counteract the EIS program, promoting cell cycle progression (Fig. 5A), despite the DNA damage induced by etoposide (Fig. S4A). However, this is a temporary effect, as CDCA2 over-expressing cells finally arrested their growth and showed SA-β-gal accumulation, just like control cells (Fig. S4B).

Etoposide provokes DNA strand breaks that induce senescence through the activation of the p53 pathway [13]. Furthermore, it has been demonstrated that, in human non-tumorigenic cells (MCF10A), the over-expression of CDCA2 attenuates DDR activation induced by etoposide treatment by recruiting PP1γ phosphatase to chromatin at damaged sites and causing the dephosphorylation of activated ATM [34]. This phenomenon could also explain the resistance to EIS showed by IMR90 cells over-expressing CDCA2. Therefore, we measured ATM activation in CDCA2 over-expressing cells 24 h after EIS induction.

As shown in Fig. 5B and 5C, the over-expression of CDCA2 is accompanied by a reduction of ATM activation. This results in a reduced activation of p53 and a consequent weak expression of the cyclin-dependent kinase inhibitor p21Cip1, which can explain the sustained cell proliferation observed despite etoposide treatment.

All these data demonstrate that also in primary human cells CDCA2 over-expression is able to antagonize the activation of ATM-dependent signal transduction modulating DDR sensitivity, thus preventing the arrest of cell-cycle progression of premature senescence via decreased expression of CDKIs.

## Discussion

In this study, with the intent to find novel direct targets of two specific SAmiRs previously associated to the induction and maintenance of cellular senescence [17], we took advantage of gene expression data available in literature to select by a comparative analysis and validate a subset of genes, whose expression was strongly reduced in replicative and stress-induced senescent cells. Among these genes we found that CDCA2, a specific nuclear regulatory subunit of protein phosphatase 1 γ (PP1γ), and ID4, a member of a family of helix-loop-helix transcription factors, are direct targets of SAmiR-494 and SAmiR-486-5p, respectively.

In our study, the down-regulation in HDFs of CDCA2, ID4 or both fails to cause a massive cell cycle arrest typical of premature senescence, that instead occurs after the over-expression of their negative regulators, SAmiR-494 or SAmiR-486-5p. Nevertheless, the ectopic expression of CDCA2, but not of ID4, was able to partially counteract the progression of EIS by the reduction of ATM activation, thus avoiding the cell cycle arrest caused by the activation of p53 and of DNA damage checkpoints after etoposide treatment. This would make cells less sensitive to DNA damage. Thus, it can be speculated that the down-regulation of CDCA2 expression that occurs in HDFs during cellular senescence could contribute to the accumulation of DNA damage that, in turn, sustains, rather than provokes, the senescence program.

CDCA2, also known as Repo-Man, is involved in cell cycle regulation [35–38), as well as in PP1γ-dependent essential DDR regulation [34]. Our data are in accordance with previous findings from Peng and colleagues, reporting that CDCA2 recruits PP1γ to chromatin to antagonize activation of ATM-dependent signal transduction in pre-malignant (not cancerous) cells [34].

Moreover, CDCA2-dependent DDR regulation is strengthened by CDCA2 over-expression during cancer progression, resulting in reduced DDR sensitivity. CDCA2 is frequently over-expressed in many tumor cells, as neuroblastoma, melanoma, breast cancer and in oral squamous cell carcinoma [39]. In particular, experiments on oral cancer cell lines showed that suppression of CDCA2 expression with shRNA significantly inhibits cellular proliferation, by arresting cell-cycle progression at the G1 phase through activation of the DDR *in vitro*, thus suggesting that up-regulation of CDCA2 in tumor cells might prevent the arrest of cell-cycle progression, via decreased expression of CDKIs and regulation of the DDR.

However, in our study CDCA2 over-expressing cells finally encounter a senescent growth arrest. This behavior might be explained by the fact that the reduction of p53 and p21 activation is only partial. Other mechanisms, parallel to ATM activation, may be involved in supporting the phosphorylation of p53.

Concerning ID4, it is an helix-loop-helix transcription factor that, having lost the basic DNA-binding domain, acts as dominant-negative regulator by forming inactive heterodimeric complexes with other helix-loop-helix transcription factors [40]. It performs different regulatory functions mainly during embryogenesis, being involved in the neural stem cell, oligodendrocyte and astrocyte differentiation.

Like CDCA2, ID4 is expressed in several tumors where it seems to play a key role in cellular transformation, immortalization, invasion, and in the metastatic process [41]–[42]. Thus, a decrease in ID4 expression levels could be one of the mechanisms adopted by cellular senescence to antagonize the tumor development in pre-cancerous cells. Nevertheless, the expression of ID4 is epigenetically silenced in prostate cancer and, very recently, it has been demonstrated that ectopic over-expression of ID4 promotes cellular senescence in prostate cancer cell line DU145 by increasing the expression of p16, p21, p27, E-cadherin and vimentin, but down-regulating E2F1 expression. ID4 also potentiated the effect of doxorubicin induced senescence and apoptosis [43]. These findings suggest that the role of ID4 in cellular senescence could be strictly dependent on tissue or cell types.

In conclusion, we have identified CDCA2 and ID4 as new direct targets of two different miRs, whose up-regulation in HDFs is correlated to the induction of cellular senescence. As expected, both targets are down-regulated during replicative- or stress-induced senescence, but cannot induce senescence when silenced in young HDFs. Nevertheless, for CDCA2, our results are in accordance with previous findings in pre-malignant (not cancerous) or tumor cells, in which the levels of CDCA2 determine the activation threshold of the DNA damage checkpoint. Thus, our results indicate that CDCA2 sets the threshold for checkpoint activation also in HDFs.

## Supporting Information

Figure S1 **Characterization of senescent IMR90 cells.** A) Characterization of replicative senescent IMR90 cells. Primary human fibroblasts IMR90 were grown in DMEM supplemented with 10% (v/v) fetal bovine serum and 1% penicillin/streptomycin for some months, until PDL 58. B) Characterization of cellular senescence induced by etoposide treatment. PDL 33 IMR90 cells were exposed to etoposide 20 µM for 24 h and then cultured for 10 days before harvesting. C) Characterization of cellular senescence induced by DEM treatment. PDL 33 IMR90 cells were chronically exposed to DEM 150 µM on alternate days for 10 days before harvesting. In all cases, cellular senescence was assessed by SA-β-gal staining and gene expression profile [4]. For SA-β-gal staining, representative images of control and senescent cells are reported. For each experiment, at least 300 cells were counted. For gene expression profiling, quantitative Real-Time PCR analysis of cyclin A (CyclA), thymidylate synthase (THY), cyclin-selective ubiquitin carrier protein (UBI), interleukin-6 (IL6), cyclin-dependent kinase inhibitors p21^Cip1^ (p21) and p16^INK4A^ (p16) mRNAs are showed. Results are the mean of triplicate determinations.
(TIFF)

Figure S2 **Quantification of SAmiR expression levels after their ectopic over-expression.** The expression levels of SAmiR-494 and SAmiR-486-5p were measured by Real Time PCR in PDL 33 IMR90 cells transfected with 100 nM pre-miR. The microRNA relative expression was calculated by assigning the arbitrary value 1 to the amount found in control pre-miR transfected cells. SD is used to refer to the values obtained in 3 different experiments. In all cases, the difference was significant (p < 0.01). A) Data refers to SAmiR’s over-expression of Figure 3A; B) data refers to SAmiR’s over-expression of Figure 3D and 3E.
(TIFF)

Figure S3 **A) Luciferase assay of unvalidated SAmiR predicted target genes.** Luciferase constructs bearing the normal 3’UTRs of FOXM1, NUSAP1 (predicted targets of SAmiR-494) and BUB1b (predicted target of SAmiR-486-5p) were transfected in HEK293 cells, together with the specific SAmiR pre-miR, control pre-miR or an unrelated pre-miR. Luciferase levels were reported as fold changes compared to the values measured in control pre-miR transfected cells, after normalization with Renilla luciferase activity. **B) SAmiR’s target knock-down by siRNAs.** The expression levels of CDCA2 and ID4 were measured by Real Time PCR in PDL 33 IMR90 cells transfected with 100 nM siRNAs, individually or as a mix. The mRNA relative expression was calculated by assigning the arbitrary value 1 to the amount found in scramble-transfected cells. SD is used to refer to the values obtained in 3 different experiments. In all cases, the difference was significant (p < 0.05).
(TIFF)

Figure S4 **A) Etoposide treatment of IMR90 cells over-expressing CDCA2 induces γH2AX foci.** PDL 33 IMR90 cells were transfected with control CMV-NEO vector or with CMV-CDCA2. After 24 h, transfected cells were treated with 20 µM etoposide. Cells were fixed and examined by immunofluorescence for a-H2AX phosphorylated on Ser139 (γH2AX) at 0, 6 or 18 hours after treatment. Coverslips were washed and incubated with Alexa-488 Goat anti-rabbit antibody and counterstained with DAPI. Counts of at least 300 cells were averaged and expressed as percent of cells positive to the presence of γH2AX foci ± SD. **B) Etoposide treatment of IMR90 cells over-expressing CDCA2 induces premature senescence.** PDL33 IMR90 cells were transfected with control vector or a vector containing the coding sequence of human CDCA2 gene. After 24 h, transfected cells were treated with 20 µM etoposide for 24 h and then were subcultivated for 10 days before harvesting. Cellular senescence was assessed by SA-β-gal staining. At least 300 cells were counted. Representative images of control and senescent cells are showed.
(TIFF)

Table S1 **Genes whose expression is down-regulated during senescence of human diploid fibroblasts.**
(PDF)

Table S2 **Primer pairs for Real Time PCR.**
(PDF)

Table S3 **Primer pairs for plasmid construction.**
(PDF)
