# Supplementary figures and images for: Comparative Analysis of Gene Expression Data Reveals Novel Targets of Senescence-Associated microRNAs
Source: PLoS One. 2014 Jun 6;9(6):e98669. doi: 10.1371/journal.pone.0098669 (PMC4048207; doi:10.1371/journal.pone.0098669)

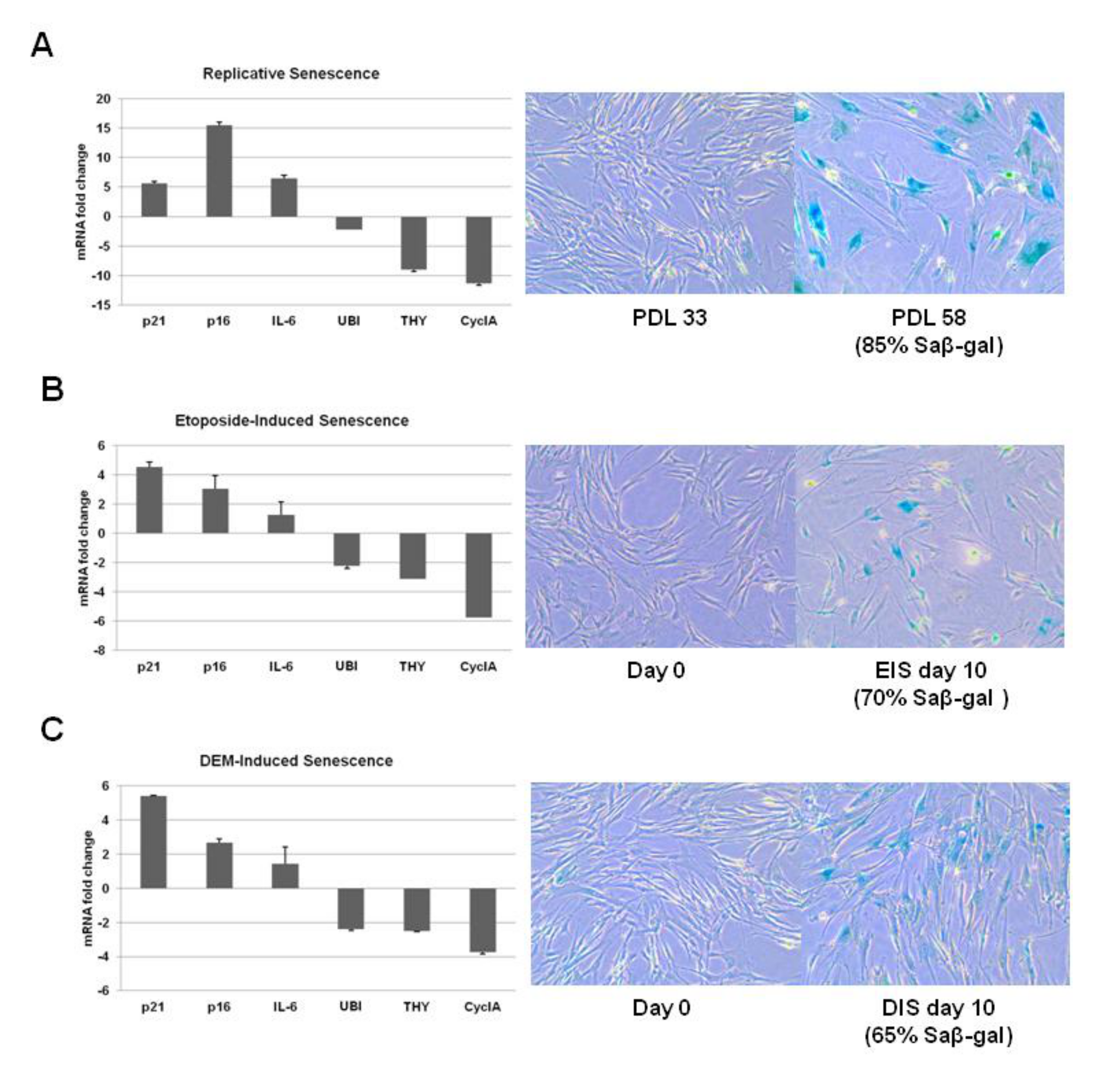

Supplement: Figure S1 — Characterization of senescent IMR90 cells. A) Characterization of replicative senescent IMR90 cells. Primary human fibroblasts IMR90 were grown in DMEM supplemented with 10% (v/v) fetal bovine serum and 1% penicillin/streptomycin for some months, until PDL 58. B) Characterization of cellular senescence induced by etoposide treatment. PDL 33 IMR90 cells were exposed to etoposide 20 µM for 24 h and then cultured for 10 days before harvesting. C) Characterization of cellular senescence induced by DEM treatment. PDL 33 IMR90 cells were chronically exposed to DEM 150 µM on alternate days for 10 days before harvesting. In all cases, cellular senescence was assessed by SA-β-gal staining and gene expression profile [4]. For SA-β-gal staining, representative images of control and senescent cells are reported. For each experiment, at least 300 cells were counted. For gene expression profiling, quantitative Real-Time PCR analysis of cyclin A (CyclA), thymidylate synthase (THY), cyclin-selective ubiquitin carrier protein (UBI), interleukin-6 (IL6), cyclin-dependent kinase inhibitors p21Cip1 (p21) and p16INK4A (p16) mRNAs are showed. Results are the mean of triplicate determinations. (TIFF) [file pone.0098669.s001.tif]

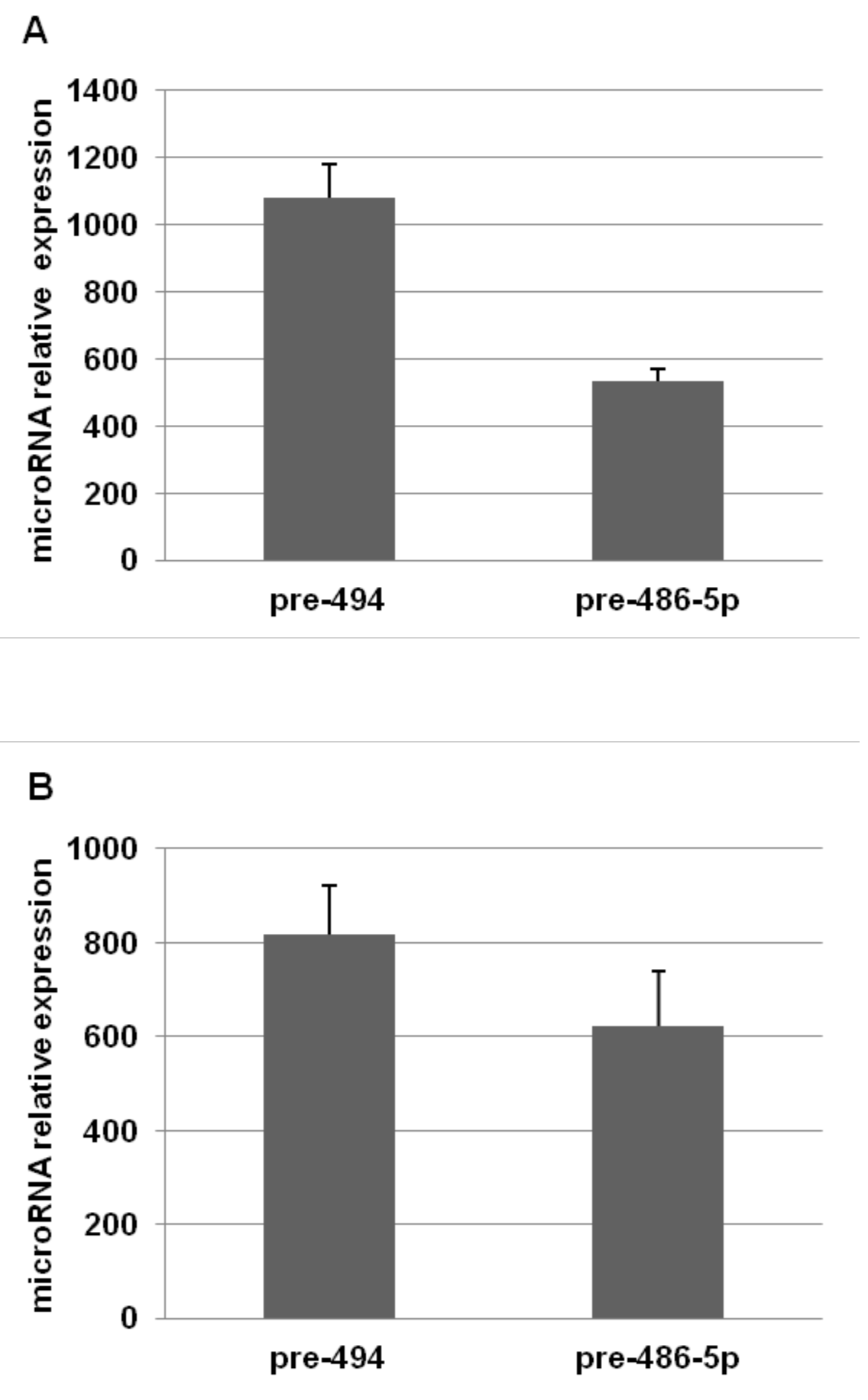

Supplement: Figure S2 — Quantification of SAmiR expression levels after their ectopic over-expression. The expression levels of SAmiR-494 and SAmiR-486-5p were measured by Real Time PCR in PDL 33 IMR90 cells transfected with 100 nM pre-miR. The microRNA relative expression was calculated by assigning the arbitrary value 1 to the amount found in control pre-miR transfected cells. SD is used to refer to the values obtained in 3 different experiments. In all cases, the difference was significant (p < 0.01). A) Data refers to SAmiR’s over-expression of Figure 3A; B) data refers to SAmiR’s over-expression of Figure 3D and 3E. (TIFF) [file pone.0098669.s002.tif]

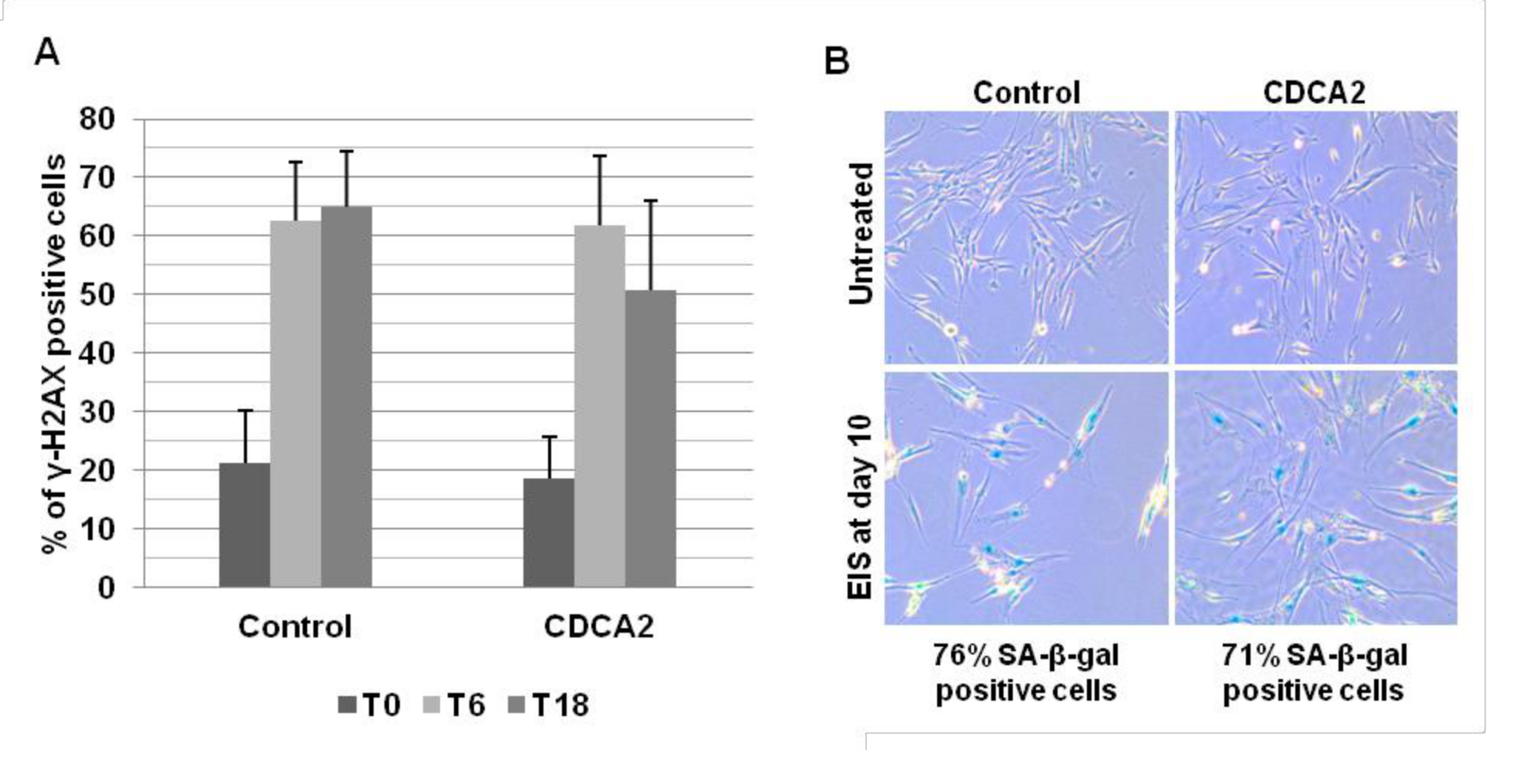

Supplement: Figure S4 — A) Etoposide treatment of IMR90 cells over-expressing CDCA2 induces γH2AX foci. PDL 33 IMR90 cells were transfected with control CMV-NEO vector or with CMV-CDCA2. After 24 h, transfected cells were treated with 20 µM etoposide. Cells were fixed and examined by immunofluorescence for a-H2AX phosphorylated on Ser139 (γH2AX) at 0, 6 or 18 hours after treatment. Coverslips were washed and incubated with Alexa-488 Goat anti-rabbit antibody and counterstained with DAPI. Counts of at least 300 cells were averaged and expressed as percent of cells positive to the presence of γH2AX foci ± SD. B) Etoposide treatment of IMR90 cells over-expressing CDCA2 induces premature senescence. PDL33 IMR90 cells were transfected with control vector or a vector containing the coding sequence of human CDCA2 gene. After 24 h, transfected cells were treated with 20 µM etoposide for 24 h and then were subcultivated for 10 days before harvesting. Cellular senescence was assessed by SA-β-gal staining. At least 300 cells were counted. Representative images of control and senescent cells are showed. (TIFF) [file pone.0098669.s004.tif]
