# Supplementary material for: Comparative Analysis of Gene Expression Data Reveals Novel Targets of Senescence-Associated microRNAs
Source: PLoS One. 2014 Jun 6;9(6):e98669. doi: 10.1371/journal.pone.0098669 (PMC4048207; doi:10.1371/journal.pone.0098669)
Supplement: Table S1 — Genes whose expression is down-regulated during senescence of human diploid fibroblasts. (PDF) [file pone.0098669.s005.pdf]

Table S1: Genes whose expression is down-regulated during senescence of human diploid fibroblasts.

Gene Symbol	Gene Name	ID number	Molecular Function	Biological Process
ANLN	Anillin	NM_018685	actin binding	regulation of exit from mitosis/septin assembly and septum formation
ASNS	Asparagine synthetase	NM_001673	asparagine synthase (glutamine-hydrolyzing) activity/ligase activity	asparagine biosynthesis/glutamine metabolism
ASPM	Abnormal spindle-like microcephaly-associated protein	NM_018136	calmodulin binding	developmental growth/negative regulation of neuron differentiation/mitosis/oogenesis/positive regulation of neuroblast proliferation
ATAD2	ATPase family, AAA domain containing 2	NM_014109	ATP binding/ATPase activity	regulation of transcription, DNA-dependent
BIRC5	Survivin	NM_001168	Ran GTPase binding/chaperone binding/ion binding/microtubule binding/cysteine-type endopeptidase inhibitor activity	negative regulation of apoptotic process/cell division/mitosis/protein phosphorilation/transcription, DNA-dependent
BRCA2	Breast cancer 2	NM_000059	H3 histone acetyltransferase activity/gamma-tubulin binding/protein binding/single strand DNA binding	DNA damage response, signal transduction by p53 class mediator resulting in transcription of p21 class mediator/DNA repair/brain development/cell aging/regulation of mitotic cell cycle/regulation of cytokinesis
BUB1	Budding Uninhibited by Benzimidazoles 1 homolog (yeast)	NM_004336	ATP binding/protein binding/protein kinase activity	apoptosis/cell proliferation/mitosis/spindle assembly checkpoint
BUB1B	Budding Uninhibited by Benzimidazoles 1 homolog Beta (yeast)	NM_001211	ATP binding/protein binding/protein kinase activity	anaphase-promoting complex-dependent proteasomal ubiquitin-dependent protein catabolic process/apoptotic process/cell cycle/cell division/cell proliferation
BUB3	Budding Uninhibited by Benzimidazoles 3 homolog (yeast)	NM_004725	protein binding	mitotic spindle checkpoint/cell proliferation
CCL20	chemokine (C-C motif) ligand 20	NM_004591	chemokine activity	cell-cell signaling/chemokinesis/chemotaxis/defence response to bacterium/immune response/inflammatory response/signal transduction
CCNA2	Cyclin A2	NM_001237	protein kinase binding	mitotic G2 checkpoint/regulation of CDK activity/cytokinesis
CCNB2	Cyclin B2	NM_004701	protein kinase binding	G2/M transition of mitotic cell cycle/T cell homeostasis/cell division/growth/in utero embryonic development
CCNE2	Cyclin E2	NM_057749	protein kinase binding	cell cycle checkpoint/regulation of CDK activity/cytokinesis
CDC2	Cell division cycle 2	NM_001786	cyclin-dependent protein kinase activity/ATP binding/transferase activity	start control point of mitotic cell cycle/protein amino acid phosphorylation/cytokinesis
CDC25A	Cell division cycle 25A	NM_001789	protein binding/protein tyrosine phosphatase activity	DNA replication/mitosis/cell division/cell proliferation/cellular response to UV
CDC25B	Cell division cycle 25B	NM_021874	protein tyrosine phosphatase activity	positive regulation of cell proliferation/mitosis/regulation of cell cycle
CDCA2	Cell division cycle associated 2	NM_152562	protein binding	cell division/mitosis
CDCA4	Cell division cycle associated 4	NM_017955	null	null
CDCA7	Cell division cycle associated 7	NM_031942	null	regulation of cell proliferation/regulation of transcription, DNA dependent

CDKN2C	Cyclin-dependent kinase inhibitor 2C (p18)	NM_001262	cyclin-dependent protein serine/threonine kinase inhibitor activity/protein binding	G1/S transition of mitotic cell cycle/cell cycle arrest/negative regulation of proliferation/negative regulation of phosphorilation/oligodendrocyte differentiation
CDKN3	Cyclin-dependent kinase inhibitor 3	NM_005192	proteine binding/proteine kinase activity	G1/S transition of mitotic cell cycle/cell cycle arrest/negative regulation of proliferation/peptidyl-tyrosine dephosphorylation
CENPE	Centromere protein E (312kD)	NM_001813	ATP binding/kinetochore binding/microtubule motor activity	cell division/blood coagulation/kinetochore assembly/microtubule-based movement/mitosis/multicellular organism development
CENPF	Centromere protein F, 350/400ka (mitosin)	NM_016343	chromatin binding/dynein binding/protein C-terminus binding/transcription factor binding	DNA replication/mitotic cell cycle/cell differentiation/cell proliferation/cell division/chromosome segregation/protein transport/kinetochore assembly/response to drug
CENPH	Centromere protein H	NM_022909	kinetochore binding/protein binding	cell division/chromosome segregation/kinetochore organization/mitotic cell cycle/nucleosome assembly
CEP55	Centrosomal protein 55kDa	NM_018131	protein binding	cytokinesis/mitosis
CKAP2	Cytoskeleton associated protein 2	AK022982	null	apoptotic process/mitotic cytokinesis/negative regulation of microtubule depolymerization/positive regulation of transcription from RNA polymerase II promoter
CKS2	CDC28 protein kinase 2	NM_001827	cyclin-dependent protein kinase activity	regulation of CDK activity/cell cycle/cytokinesis
COL15A1	Collagen, type XV, alpha 1	NM_001855	extracellular matrix structural constituent	angiogenesis/cell adhesion/cell differentiation/collagen catabolic process/extracellular matrix organization/signal transduction
COLEC12	Collectin sub-family member 12	NM_030781	galactose binding/low-density lipoprotein particle binding/metal ion binding/scavenger receptor activity	carbohydrate mediated signaling/defense response/innate immune response/phagocytosis, recognition
CSNK1G1	casein kinase 1, gamma 1	NM_014736	ATP binding/glycoprotein binding/magnesium ion binding/phosphoprotein binding	Wnt receptor signaling pathway/protein autophosphorilation
CTNNAL1	Catenin, alpha-like 1	NM_003798	cadherin binding/structural molecule activity	Rho protein signal transduction/cell adhesion
CYP1B1	Cytochrome P450, subfamily I , polypeptide 1	NM_000104	aromatase activity/electron carrier activity/heme binding/iron ion binding/monooxygenase activity/oxygen binding	angiogenesis/arachidonic acid metabolic process/cellular aromatic metabolic process/oxidation-reduction process/epoxygenase P450 pathway
DDX18	DEAD (Asp-Glu-Ala-Asp) box polypeptide 18	NM_006773	ATP dependent RNA helicase activity/ATP binding/RNA binding	null
DEK	DEK oncogene (DNA binding)	NM_003472	DNA binding/histone binding	chromatin modification/redulation of double-strand berak repair/signal transduction
DEPDC1	DEP domain containing 1	NM_017779	GTPase activator activity/protein binding	intracellular signal transduction/negative regulation of transcription, DNA dependent
DHFR	Dihydrofolate reductase	NM_000791	dihydrofolate reductase activity/oxidoreductase activity	glycine biosynthesis/one-carbon compound metabolism/nucleotide biosynthesis
DLGAP5	Discs Large Associated Protein 5 (Drosophila)	NM_014750	phosphoprotein phosphatase activity/protein binding	cell proliferation/cell-cell signaling/M phase of mitotic cell cycle
DONSON	Downstream neighbor of SON	NM_017613	null	null
DTYMK	Deoxythymidylate kinase	NM_012145	thymidylate kinase activity/ATP binding/kinase activity/transferase activity	cell cycle/DNA metabolism/dTDP biosynthesis/dTTP biosynthesis/nucleotide biosynthesis

EIF4EBP1	Eukaryotic translation initiation factor 4E binding protein 1	NM_004095	protein biosynthesis inhibitor activity	regulation of translation/regulation of protein biosynthesis
ENO1	Enolase 1, (alpha)	NM_001428	phosphopyruvate hydratase activity/transcription co-repressor activity/DNA binding/transcription factor activity	negative regulation of transcription from Pol II promoter
ENPP2	Ectonucleotide pyrophosphatase/phosphodiesterase 2 (autotaxin)	NM_006209	phosphodiesterase I activity/nucleotide diphosphatase activity/nucleic acid binding/transcription factor binding/endonuclease activity/hydrolase activity	nucleotide metabolism/phosphate metabolism/G-protein coupled receptor protein signaling pathway/chemotaxis/cell motility
EXO1	Exonuclease 1	NM_003686	5'-3' exodeoxyribonuclease activity/DNA binding/flap endonuclease activity/metal ion binding/protein binding/ribunuclease H activity/structure-specific DNA binding	DNA recombination/DNA repair/isotype switching/mismatch repair
FBL	Fibrillarin	NM_001436	RNA binding	rRNA processing
FBXO5	F-box only protein 5	NM_012177	metal ion binding/protein kinase binding	cell division/mitosis/microtubule polymerization/negative regulation of meiosis/negative regulation of ubiquitin-protein ligase activity involved in mitotic cell cycle/oocyte maturation/vesicle organization
FOXM1	Forkhead box M1	NM_021953	DNA binding/transcription factor binding	DNA damage response, signal transduction by p53 class mediator resulting in transcription of p21 class mediator/DNA repair/negative regulation of cell aging/regulation of transcription/regulation of cell proliferation/regulation of cell growth/tissue development
FST	Follistatin	NM_006350	activin inhibitor activity	development/negative regulation of follicle-stimulating hormone secretion
GLUL	Glutamate-Ammonia Ligase	NM_002065	glutamate-ammonia ligase activity/ligase activity	regulation of neurotransmitter levels/glutamine biosynthesis/nitrogen fixation
GMNN	Geminin	NM_015895	histone deacetylase binding/protein binding/repressing transcription factor binding	negative regulation of DNA replication/cell cycle arrest/cell cycle
GPC6	Glypican 6	NM_005708	transmembrane receptor activity	cell growth and/or maintenance/development
GTSE1	G-2 and S-phase expressed 1	NM_016426	molecular_function unknown	G2 phase of mitotic cell cycle/DNA damage response, signal transduction by p53 class mediator resulting in cell cycle arrest/microtubule-based process
H2AFX	H2A histone family, member X	NM_002105	DNA binding	nucleosome assembly/chromosome organization and biogenesis (sensu Eukarya)
H2AFZ	H2A histone family, member Z	NM_002106	DNA binding/protein heterodimerization activity	nucleosome assembly
H3F3B	H3 histone family, member 3B	NM_005324	DNA binding	nucleosome assembly/chromosome organization and biogenesis (sensu Eukarya)
HAT1	Histone acetyltransferase 1	NM_003642	histone acetyltransferase activity/acyltransferase activity/protein binding/transferase activity	internal protein amino acid acetylation/DNA packaging
HELLS	Helicase, lymphoid-specific	NM_018063	ATP binding/DNA binding/helicase activity	cell division/centromeric heterochromatin assembly/lymphocyte proliferation/maintenance of DNA methylation
HMGB1	High-mobility group box 1	NM_002128	single-stranded DNA binding	regulation of transcription, DNA-dependent/DNA unwinding

HMGB2	High-mobility group box 2	NM_002129	single-stranded DNA binding	DNA ligation involved in DNA repair/DNA topological change/V(D)J recombination/apoptotic process/cell chemotaxis/nucleosome assembly
HMGN2	High-mobility group nucleosomal binding domain 2	NM_005517	nucleosomal DNA binding	chromatin organization/regulation of transcription, DNA-dependent
HNRPA2B1	Heterogeneous nuclear ribonucleoprotein A2/B1	NM_031243	heterogeneous nuclear ribonucleoprotein/RNA binding	RNA processing
HNRPAB	Heterogeneous nuclear ribonucleoprotein A/B	NM_031266	RNA binding	epithelial to mesenchymal transition/positive regulation of transcription, DNA-dependent
ID4	Inhibitor of DNA binding 4	NM_001546	transcription co-repressor activity	regulation of transcription from Pol II promoter
ITGB3BP	Integrin beta 3 binding protein (beta3-endonexin)	NM_014288	protein C-terminus binding/protein binding/signal transducer activity	cell adhesion/signal transduction
KDM1A	Lysine (K)-specific demethylase 1A	NM_015013	RNA polymerase II repressing trascription factor activity/demethylase activity/oxidoreductase activity	blood coagulation/cell proliferation/histone demethylation/negative regulation of DNA binding/negartive regulation of DNA damage response
KHDRBS1	KH domain containing, RNA binding, signal transduction associated 1	NM_006559	mRNA binding/single-stranded DNA binding/single-stranded RNA binding/DNA binding	G1/S transition of mitotic cell cycle/cell cycle arrest/mRNA processing/RAS protein signal transduction/cell proliferation/signal transduction
KIF11	Kinesin family member 11	NM_004523	microtubule motor activity/ATP binding	mitotic spindle assembly
KIF20B	Kinesin family member 20B	NM_016195	ATP binding/ATPase activity/WW domain binding/microtubule binding/microtubule motor activity	ATP catabolic process/mitosis/cell cycle arrest/microtubule-based movement
KIF22	Kinesin family member 22	NM_007317	microtubule motor activity/DNA binding	mitosis
KIF23	Kinesin family member 23	NM_004856	microtubule motor activity/adenosinetriphosphatase activity	mitotic spindle elongation
KITLG	KIT ligand	NM_000899	cytokine activity/growth factor activity	cell adhesion/cell proliferation/embryonic hemopoiesis/germ cell programmed cell death/signal transduction
KNTC1	Kinetochore associated 1	NM_014708	protein binding	cell division/mitosis/protein complex assembly
KPNA2	Karyopherin alpha 2 (RAG cohort 1, importin alpha 1)	NM_002266	nuclear localization sequence binding/protein transporter activity/protein binding	regulation of DNA recombination/G2 phase of mitotic cell cycle/spindle pole body and microtubule cycle (sensu Saccharomyces)/NLS-bearing substrate-nucleus import/intracellular protein transport/DNA metabolism
LBR	Lamin B receptor	NM_002296	lamin/chromatin binding/lamin binding/DNA binding/receptor activity	cholesterol biosynthetic process
LMNB2	Lamin B2	NM_032737	structural molecule activity	S-specific transcription in mitotic cell cycle
MAD2L1	MAD2-like 1	NM_002358	protein binding/protein homodimerization activity	cell division/mitotic cell cycle/negative regulation of apoptotic process
MAPK13	MAP Kinase 13	NM_002754	MAP kinase activity/ATP binding/protein serine/threonine kinase activity/MP kinase activity/transferase activity	protein kinase cascade/cell cycle/response to stress/protein amino acid phosphorylation/regulation of translation/signal transduction/antimicrobial humoral response (sensu Vertebrata)
MARCKS	Myristoylated alanine-rich protein kinase C substrate	NM_002356	calmodulin binding/actin cross-linking activity	cell motility
MCM3	Minichromosome maintenance deficient 3 (S. cerevisiae)	NM_002388	DNA dependent ATPase activity/ATP binding/DNA binding	cell cycle/DNA replication/DNA replication initiation/regulation of transcription, DNA-dependent

MCM4	Minichromosome maintenance deficient 4 (S. cerevisiae)	NM_182746	ATP binding/contributes to ATP-dependent DNA helicase activity/protein binding/single-strand DNA binding	DNA replication/mitotic cell cycle
MCM5	Minichromosome maintenance deficient 5 (S. cerevisiae)	NM_006739	DNA dependent ATPase activity/ATP binding/DNA binding	DNA replication/DNA replication initiation/regulation of transcription, DNA-dependent/regulation of cell cycle
MEIS1	Meis homeobox 1	NM_002398	RNA polymerase II distal enhancer sequence-specific DNA binding transcription factor activity/protein binding	angiogenesis/definitive hemopoiesis/negative regulation of neuron differentiation
MELK	Maternal Embryonic Leucine zipper Kinase	NM_014791	ATP binding/protein serine/threonine kinase activity/transferase activity	protein amino acid phosphorylation
MKI67	Antigen identified by monoclonal antibody Ki-67	NM_002417	ATP binding/protein binding	DNA metabolic process/cell proliferation/meiosis/cellular response to heat/organ regeneration
MTHFD2	Methylenetetrahydrofolate dehydrogenase (NADP+ dependent) 2, methenyltetrahydrofolate cyclohydrolase	X16396	magnesium ion binding/methenyltetrahydrofolate cyclohydrolase activity/methenyltetrahydrofolate dehydrogenase activity	folic acid-containing compound biosynthetic process/one carbon metabolic process/response to heat
MYH10	Myosin Heavy Chain 10, non muscolar	NM_005964	actin binding/ATP binding/motor activity/calmodulin binding	cytokinesis
NASP	Nuclear autoantigenic sperm protein	NM_002482	hsp90 protein binding/histone binding	DNA packaging/spermatogenesis
NEK2	NIMA-related kinase 2	NM_002497	ATP binding/protein serine/threonine kinase activity/transferase activity	regulation of mitosis/meiosis/protein amino acid phosphorylation/cytokinesis
NETO2	neuropilin (NRP) and tolloid (TLL)-like 2	NM_018092	null	null
NOL5A	Nucleolar protein 5A (56kD with KKE/D repeat)	NM_006392	RNA binding/DNA binding	rRNA processing/regulation of transcription, DNA-dependent
NQO1	NAD(P)H dehydrogenase, quinone 1	NM_000903	NAD(P)H dehydrogenase (quinone) activity/cytochrome-b5 reductase activity/oxidoreductase activity	nitric oxide biosynthesis/response to toxin/synaptic transmission, cholinergic/xenobiotic metabolism/electron transport
NREP (C5orf13)	Neuronal Regeneration Related Protein	NM_004772	protein binding	axon regeneration/regulation of neuron differentiation/regulation of transforming growth factor beta receptor signaling pathway
NRP1	Neuropilin 1	NM_003873	coreceptor activity/cytokine binding/heparin binding/metal ion binding/semaphorin receptor activity/VEGF-activated receptor activity	axon guidance/cell adhesion/cell-cell signaling/endothelial cell chemotaxis/organ morphogenesis
NUF2	NDC80 kinetochore complex component, homolog (S. cerevisiae)	NM_031423	protein binding	cell division/mitosis
NUSAP1	Nucleolar and spindle associated protein 1	NM_016359	DNA binding/microtubule binding	positive regulation of mitosis
PA2G4	Proliferation-associated 2G4, 38kD	NM_006191	methionyl aminopeptidase activity/hydrolase activity	cell cycle arrest/proteolysis and peptidolysis/cell proliferation
PBK	PDZ binding kinase	NM_018492	ATP binding/protein binding/protein serine-threonine kinase activity	mitosis/protein phosphorilation
PCNA	Proliferating cell nuclear antigen	NM_002592	DNA binding/DNA polymerase processivity factor activity	regulation of DNA replication/DNA replication/DNA repair/cell proliferation/regulation of cell cycle

PCOLCE	Procollagen C-endopeptidase enhancer	NM_002593	collagen binding/heparin binding/peptidase activator activity	multicellular organismal development/positive regulation of peptidase activity/proteolysis
PHGDH	Phosphoglycerate dehydrogenase	BC011262	NAD binding/electron carrier activity/phosphoglycerate dehydrogenase activity	cellular amino acid biosynthetic process/glial cell development
PLK1	Polo-Like Kinase 1	NM_005030	ATP binding/anaphase-promoting complex binding/kinase activity/microtubule binding	G2 DNA damage checkpoint/mitosis/cytokinesis/cell proliferation/positive regulation of proteolysis
POLE	DNA Polimerase epsilon	NM_006231	nucleotide binding/3'-5' exonuclease activity/DNA-directed DNA polymerase activity/DNA binding/transferase activity	DNA replication/DNA repair
PRC1	Protein regulator of cytokinesis 1	NM_003981	protein binding/microtubule binding/	cytokinesis/mitotic spindle elongation
PRIM1	Primase, polypeptide 1	NM_000946	DNA primase activity/metal ion binding	DNA replication/DNA strand elongation involved in DNA repair/mitotic cell cycle/telomere maintenance
PTMA	Prothymosin, alpha	NM_002823	null	transcription, DNA-dependent
PTTG1	Pituitary Tumor Transforming 1	NM_004219	transcription factor activity	spermatogenesis/oncogenesis/transcription from Pol II promoter
RAD51	RAD51	NM_002875	DNA dependent ATPase activity/damaged DNA binding/nucleotide binding/ATP binding	mitotic recombination/meiotic recombination/DNA repair
RAD51AP1	RAD51-interacting protein	NM_006479	RNA binding/DNA binding/protein binding	DNA repair
RBBP8	Retinoblastoma binding protein 8	NM_002894	RNA polymerase II repressing trascription factor activity/damage DNA binding/protein binding	DNA repair/cell division/blastocyst hatching/meiosis/mitosis/negative regulation of transcription from RNA polymerase II promoter
RCC1	Regulator of chromosome condensation 1	NM_001269	Ran guanyl-nucleotide exchange factor activity/chromatin binding/histone binding	cell division/mitosis/viral process
RFC2	Replication factor C (activator 1) 2 (40kD)	NM_002914	nucleotide binding/ATP binding/DNA binding	DNA replication
RFC3	Replication factor C (activator 1) 3 (40kD)	NM_002915	nucleotide binding/DNA-directed DNA polymerase activity/enzyme activator activity/DNA binding	DNA replication
RFC4	Replication factor C (activator 1) 4 (37kD)	NM_002916	nucleotide binding/DNA-directed DNA polymerase activity/enzyme activator activity/DNA binding	DNA replication
RPL36A	Ribosomal protein L36a	NM_021029	structural constituent of ribosome	RNA metabolic process/SRP-dependent cotraslational protein targeting to membrane/gene expression/ protein metabolism/translation/viral process
RPL5	Ribosomal protein L5	NM_000969	structural constituent of ribosome/rRNA binding/5S rRNA binding	protein biosynthesis
RRM2	Ribonucleotide reductase M2 polypeptide	NM_001034	ribonucleoside-diphosphate reductase activity/oxidoreductase activity	DNA replication/deoxyribonucleoside diphosphate metabolism
SCARA3	CSR1 protein	NM_016240	scavenger receptor activity	UV protection/response to oxidative stress
SENP1	Sentrin/SUMO-specific protease	NM_014554	endopeptidase activity/cysteine-type peptidase activity	proteolysis and peptidolysis
SGOL2	Shugoshin-like 2 (S. pombe)	NM_152524	protein binding	cell division/meiotic sister chromatid cohesion, centromeric/mitotic cell cycle
SHCBP1	SHC SH2 domain binding protein 1	NM_024745	SH2 domain binding	fibroblast growth factor receptor signaling pathway/regulation of neural precursor cell proliferation
SMC2	Structural Maintenance of Chromosomes 2-like 1 (yeast)	NM_006444	ATP binding/ATP-binding cassette (ABC) transporter activity	mitotic chromosome condensation/mitotic chromosome segregation/transport/chromosome organization and biogenesis (sensu Eukarya)/mitosis

SMC4L1	Structural Maintenance of Chromosomes 4-like 1 (yeast)	AL136877	ATP binding/ATP-binding cassette (ABC) transporter activity/DNA supercoiling activity/protein heterodimerization activity	mitotic chromosome condensation/mitotic chromosome segregation/transport/cell cycle/chromosome organization and biogenesis (sensu Eukarya)/mitosis
SOCS2	Suppressor of cytokine signaling 2	NM_003877	JAK pathway signal transduction adaptor activity/SH3-SH2 adaptor activity/prowth hormone receptor binding/insulin-like growth factor receptor binding/prolactin receptor binding/protein binding	JAK-STAT cascade/aging/cellular response to hormone stimulus/negative regulation of apoptotic process/negative regulation of signal transduction/protein ubiquitination
TCF19	Transcription factor 19 (SC1)	NM_001077511	transcription factor activity	cell proliferation/regulation of cell cycle/regulation of transcription from Pol II promoter
TCOF1	Treacher Collins-Franceschetti syndrome 1	NM_000356	transporter activity	nucleocytoplasmic transport/skeletal development
TK1	thymidine kinase 1	NM_003258	thymidine kinase activity/ATP binding/kinase activity/transferase activity	DNA metabolism
TMPO	Thymopoietin	NM_003276	lamin/chromatin binding/lamin binding	regulation of transcription, DNA-dependent/transcription
TNC	Tenascin C	NM_002160	binding/cell adhesion receptor activity	cell adhesion
TOP2A (target noto 494)	Topoisomerase (DNA) II alpha (170kD)	NM_001067	DNA topoisomerase type II activity/ATP binding/DNA binding/isomerase activity	DNA metabolism/DNA topological change
TOPBP1	Topoisomerase (DNA) II binding protein	NM_007027	protein C-terminus binding/isomerase activity	DNA replication and chromosome cycle/DNA metabolism
TPX2	Microtubule associated, homolog (Xenopus laevis)	NM_012112	GTP binding/ATP binding	mitosis/cell proliferation
TRIP13	Thyroid hormone receptor interactor 13	NM_004237	transcription cofactor activity/adenosinetriphosphatase activity	transcription from Pol II promoter
TROAP	trophinin associated protein	NM_005480	protein binding	cell adhesion
TTK	TTK protein kinase	NM_003318	protein-tyrosine kinase activity/ATP binding/protein serine/threonine kinase activity/transferase activity	mitotic spindle assembly/mitotic spindle checkpoint/positive regulation of cell proliferation/protein amino acid phosphorylation/regulation of cell cycle
UBE2C	Ubiquitin protein E2-C	NM_007019	ATP binding/protein binding/ubiquitin-protein ligase activity/contributes to ubiquitin ligase activity	cell cycle/cell division/cyclin catabolic process/phosphatidylinositol-mediated signaling/free ubiquitin chain polymerization
USP1	Ubiquitin specific protease 1	NM_003368	ubiquitin-specific protease activity/cysteine-type endopeptidase activity/ubiquitin thiolesterase activity/hydrolase activity	ubiquitin-dependent protein catabolism
VCAN	Chondroitin sulfate proteoglycan 2 (versican)	NM_004385	sugar binding/hyaluronic acid binding/calcium ion binding	cell recognition/development/heterophilic cell adhesion
WDR76	WD repeat domain 76	NM_024908	null	null
ZNF367	Zinc finger protein 367	NM_153695	DNA binding /metal ion binding/sequence-specific DNA binding transcription factor activity	transcription, DNA-dependent
ZWINT	ZW10 interactor	NM_031423	protein N-terminus binding/protein binding	cell cycle/cell division/phosphatidylinositol-mediated signaling
