# Supplementary material for: Comparative Analysis of Gene Expression Data Reveals Novel Targets of Senescence-Associated microRNAs
Source: PLoS One. 2014 Jun 6;9(6):e98669. doi: 10.1371/journal.pone.0098669 (PMC4048207; doi:10.1371/journal.pone.0098669)
Supplement: Table S2 — Primer pairs for Real Time PCR. (PDF) [file pone.0098669.s006.pdf]

Table S2: Primer pairs for Real Time PCR		
**Gene**	**Forward**	**Reverse**
BUB1B	TAGGGCGTTTATGCAATGAG	TCGCATCTGCTTTCCTAAAGTT
BUB3	ACTCGCTGCATACGAGCGTT	TGTACCTCAGGGCTTGGGTC
CCNE2	TCCAAGAGTTTGCTTACGTCAC	GCCAGGAGATGATTGTTACAGG
CDCA2	ATGACAGACTTGACCAGAAAGGA	CCGACGTTTGGAGGACAACA
CDCA4	CTCCCTCCACGTCTTCCTC	CTGGCTGAGCAGAGGACC
CDCA7	TCCGACTCACAATCAAGGAGA	GGTAGAGCGTCAAGGGACC
DEK	TCATCGTGGAAGGCAAGAGG	TCTGCCCCTTTCCTTGTGC
DHFR	AAATGAGCTCCTTGTGGAGG	ACCTGGTTCTCCATTCCTGA
FOXM1	TGCCCAGCAGTCTCTTACCT	CTACCCACCTTCTGGCAGTC
H3F3B	CTTTCGTGGCCAGCTGTT	GTCGGAGAAGTGGCCTAAAA
HAT1	CATCCCCAAAGAGTTGATGG	GCAGTGGAGAAGAAACTGGC
ID4	CCGAGCCAGGAGCACTAGAG	CTTGGAATGACGAATGAAAACG
KHDRBS1	GCACTCGCTCTTTCAGTTTC	TTCACTCACGCCATGCAG
MKI67	CCACACTGTGTCGTCGTTTG	CCGTGCGCTCATCCATTCA
NETO2	ATGGCAAAAGAAAGTGCTGC	GAATGTGGGCAGATGAAGGT
NRP1	TTGCAGTCTCTGTCCTCCAA	GAAAAATGCGAATGGCTGAT
NUSAP1	AAACTTACAAACAACCCCATCTCC	GTTTCTTCCGTTGCTCTTCCTTT
PCOLCE	CTCCTCCGAAGGGAATGAAC	GCAGCTTGACTTTAGGCTCAG
RAD51	TATCCAGGACATCACTGCCA	GGTGAAGGAAAGGCCATGTA
RAD51AP1	TCGTCATTATCCTCACTCTCACA	CTTCTGGAAGGCAGTGATGG
RFC2	GTGAGCAGGCTAGAGGTCTTT	ATTGAGTTCCAACATGGCATC
RFC3	TGCTGAGCATCTTTGGTGAG	AACAGTGGCACAATCACAACA
SENP1	TCGCTCCATCAGCATATTCA	GCATTTCGCCTGACCATTAC
SOCS2	GGAGGACGGATGACAAAGTC	AGACACTCTCCGGACTGAGG
TMPO	CAACCATTATGTCCTGAGAGGTC	GTGGCGGAGAAATTAGTTGTGA
TNC	TTCAGCAGAATTGGGGATTT	ACCTAGGTCTCTCGCCCATC
VCAN	CACTCTTTTGCAGCCTCCTC	TCTCCCCAGGAAACTTACGA
ZNF367	AATCGCGGACAGTATCTGCT	GTGAGGACGAGGAGGAAGC
ZWINT	CAGGCCTACAGGTTCCAAGA	CTGGAAAGATGGAGGCAGC
