# Supplementary material for: Comparative Analysis of Gene Expression Data Reveals Novel Targets of Senescence-Associated microRNAs
Source: PLoS One. 2014 Jun 6;9(6):e98669. doi: 10.1371/journal.pone.0098669 (PMC4048207; doi:10.1371/journal.pone.0098669)
Supplement: Table S3 — Primer pairs for plasmid construction. (PDF) [file pone.0098669.s007.pdf]

Table S3: primer pairs for plasmid construction.

	Primer pairs for luciferase plasmid construction	
**Gene**	**Forward**	**Reverse**
NUSAP1	5'-TAATTTTTTAACATCTTGTAAATA-3'	5'-ATGACAATGAGAAAAAATTTTA-3'
CDCA2	5'-TTGACATTTCCTGCAGAGTCTGTG-3'	5'-TTTTCTATCTGAATGATGAAGAAACTTA-3'
BUB1b	5'-GCTAGGCAATCAAGTCTC-3'	5'-TTAGAAGCACAAATTTAACAG-3'
FOXM1	5'-AGCCCTGCCCTTGCCCCTGT-3'	5'-TGTCCACCTTCGCTTTTATTG-3'
ID4	5'-GCCGCGCTGTCCAGGTGT-3'	5'-TACAAGACAGAGAAATCTACT-3'
OLFM4	5'-CACTAGAGATCTAGGACAT-3'	5'-TAGAATATATAAGCATGCC-3'
	**Primer pairs for CMV-CDCA2 and CMV-ID4 plasmids construction**	
**Gene**	**Forward**	**Reverse**
CDCA2	5'-ATGGATGCCAATTCAAAAGACAAGC-3'	5'-TCACTGCTTTCTTTCTCCATTATGTTCAATC-3'
ID4	5'-ATGAAGGCGGTGAGCCCGGTGCGC-3'	5'-TCAGCGGCACAGAATGCTGTCGCC-3'
	**Oligos for point mutation of SAmiR-486-5p seed region on ID4's 3'UTR**	
sense	5'-CACCTTATCAGTTTTTAAGTAAGGGGTTTTATAGTGTAATATATACAG-3'	
antisense	5'-CTGTATATATTACACTATAAAACCCCTTACTTAAAAACTGATAAGGTG-3'	
	**Oligos for point mutation of SAmiR-494 seed region on CDCA2's 3'UTR**	
sense	5'-TCCCATTCTCTGTTCAACCTCAGTGCATCAAAAGTTCCTAATAAATAAACTC-3'	
antisense	5'-GAGTTTATTTATTAGGAACTTTTGATGCACTGAGGTTGAACAGAGAATGGGA-3'	
